# A Link Between Methylglyoxal and Heart Failure During HIV-1 Infection

**DOI:** 10.3389/fcvm.2021.792180

**Published:** 2021-12-14

**Authors:** Prasanta K. Dash, Fadhel A. Alomar, Jesse L. Cox, JoEllyn McMillan, Bryan T. Hackfort, Edward Makarov, Brenda Morsey, Howard S. Fox, Howard E. Gendelman, Santhi Gorantla, Keshore R. Bidasee

**Affiliations:** ^1^Departments of Pharmacology and Experimental Neuroscience, University of Nebraska Medical Center, Omaha, NE, United States; ^2^Department of Pharmacology and Toxicology, College of Clinical Pharmacy, Imam Abdulrahman Bin Faisal University, Dammam, Saudi Arabia; ^3^Departments of Pathology and Microbiology, University of Nebraska Medical Center, Omaha, NE, United States; ^4^Departments of Cellular and Integrative Physiology, University of Nebraska Medical Center, Omaha, NE, United States; ^5^Departments of Neurological Sciences, University of Nebraska Medical Center, Omaha, NE, United States; ^6^Departments of Environment and Occupational Health, University of Nebraska Medical Center, Omaha, NE, United States; ^7^Nebraska Redox Biology Center, Lincoln, NE, United States

**Keywords:** HIV-1, heart failure, humanized mice, methylglyoxal, echocardiography

## Abstract

Early-onset heart failure (HF) continues to be a major cause of morbidity and mortality in people living with human immunodeficiency virus type one (HIV-1) infection (PLWH), yet the molecular causes for this remain poorly understood. Herein NOD.Cg-Prkdc^scid^Il2rg^tm1Wjl^/SzJ humanized mice (Hu-mice), plasma from PLWH, and autopsied cardiac tissues from deceased HIV seropositive individuals were used to assess if there is a link between the glycolysis byproduct methylglyoxal (MG) and HF in the setting of HIV-1 infection. At five weeks post HIV infection, Hu-mice developed grade III-IV diastolic dysfunction (DD) with an associated two-fold increase in plasma MG. At sixteen-seventeen weeks post infection, cardiac ejection fraction and fractional shortening also declined by 26 and 35%, and plasma MG increased to four-fold higher than uninfected controls. Histopathological and biochemical analyses of cardiac tissues from Hu-mice 17 weeks post-infection affirmed MG increase with a concomitant decrease in expression of the MG-degrading enzyme glyoxalase-1 (Glo1). The endothelial cell marker CD31 was found to be lower, and coronary microvascular leakage and myocardial fibrosis were prominent. Increasing expression of Glo1 in Hu-mice five weeks post-infection using a single dose of an engineered AAV2/9 (1.7 × 10^12^ virion particles/kg), attenuated the increases in plasma and cardiac MG levels. Increasing Glo1 also blunted microvascular leakage, fibrosis, and HF seen at sixteen weeks post-infection, without changes in plasma viral loads. In plasma from virally suppressed PLWH, MG was also 3.7-fold higher. In autopsied cardiac tissues from seropositive, HIV individuals with low viral log, MG was 4.2-fold higher and Glo1 was 50% lower compared to uninfected controls. These data show for the first time a causal link between accumulation of MG and HF in the setting of HIV infection.

## Introduction

Modern antiretroviral drug therapies (ART) have profoundly reduced morbidities and mortality in human immunodeficiency virus type one (HIV-1) infected individuals (1, 2). However, by contemporary estimates more than 40% of persons living with HIV-1 infection (PLWH) on long-term combination ART therapies are developing early-onset heart failure (HF) (3–7). This disease which starts at least a decade earlier in PLWH compared to uninfected individuals and is independent of arteriosclerosis, and/or myocardial infarction. This HF also starts earlier in women than in men (6, 8–13). To date, pharmacological strategies to blunt/slow the development HF in PLWH remain virtually non-existent, in part because of an incomplete understanding of the mechanisms involved.

Available data suggest that the pathophysiology of early-onset HF in PLWH is multifactorial, arising from persistent systemic immune activation, elevation in inflammation and oxidative stress, off-target effects of antiretroviral drugs, alcohol, aging, illicit drug use and the composition of the gut microbiome (5, 14–22). However, specific molecular pathways by which these cues negatively impact cardiac function are not well-defined. Some investigators using transgenic rodents have suggested that the HIV-1 auxiliary proteins Nef, gp120 and Tat contribute to the early-onset HF by impairing mitochondria and contractile functions of myocytes (23–28). However, to the best of our knowledge, plasma levels of HIV viral proteins in PLWH on ART are significantly lower than that in the transgenic rodent models (29–31). Also, the low-grade systemic inflammation seen in PLWH is minimally observed in these transgenic rodents, raising concerns about the disease relevance of the latter. Whether EcoHIV mice, another model in which gp120 from HIV-1 is replaced with murine leukemia virus gp80 for cell entry, also develops HF remains unclear (32). Others have suggested that off-target effects of antiretroviral drugs, alcohol and illicit drug use, and accelerated aging could exacerbate traditional risk factors of cardiovascular diseases and HF potentiating dyslipidemia, hyperglycemia, and endothelial dysfunction (5, 14–21, 33). However, the effects of antiretroviral drugs, alcohol, and illicit drugs, and aging on cardiac function in the setting of HIV-1 infection are limited, making delineation of mechanisms that trigger HF development in HIV-1 setting challenging.

There are some studies in the literature showing that prior to the onset of antiretroviral therapy, HIV-infected individuals usually develop dyslipidemia, hypertension, and metabolic syndrome (34–38). Some ART-naive patients also developed left ventricular stiffness, suggesting that HIV infection itself could be initiating/triggering HF (39–42). HIV-1, like all RNA viruses depends on the host cells they infect for the metabolic resources needed for replication. Immunocytes (mononuclear phagocytes and lymphocytes) that express the CD4, CCR5 and CXCR4 are principal targets for HIV-1 infection (43). Following viral infection, glucose transporter 1 (GLUT1) is upregulated in immunocytes to facilitate the increase in glycolysis needed for viral replication and the release of new viruses (44–46). In addition to the two pyruvate and two ATP molecules (47), glycolysis also generates a cytotoxic byproduct, namely methylglyoxal (MG) from breakdown of triose intermediates, glyceraldehyde 3-phosphate and dihydroxyacetone phosphate (48). In healthy individuals, MG is kept low (between ~250 nM in plasma, and ~3 μM in tissues, respectively) by the actions of dual-enzyme, glyoxalase degradation system (49–51). In the first step, the rate-limiting glyoxalase-I (*GLOI*, EC4.4.1.5, Glo1) converts a hemi-thioacetal formed between MG and reduced glutathione (MG-GSH) into S,D-lactoylglutathione which is then degraded by glyoxalase-II (*GLOII*, EC3.1.2.6, Glo-II) in the presence of water into D-lactic acid and GSH (49–51).

MG is a potent activator of the inflammation transcription factor, nuclear factor kappa-light-chain-enhancer of activated B cells (NF-κB), the NLR family pyrin domain containing 3 (NRLP3) inflammasome and mitochondria production of reactive oxygen species (ROS) (52, 53). Glo1 expression is also negatively regulated by inflammation and oxidative stress (49, 54–57). Thus, as MG levels increase it can activate NF-kB and the NRLP3 inflammasome, increase oxidative stress and downregulate Glo1, resulting in accumulation of MG. At high levels MG can also diffuse from infected immunocytes into the microenvironment. Earlier we showed that acute exposure of vascular endothelial cells and cardiac myocytes to supraphysiologic levels of MG, perturb their intracellular Ca^2+^ homeostasis and increase ROS production (58, 59). Long-term exposure of vascular endothelial cells to MG also diminished their responses to vasodilators and expression of tight junction proteins, established causes of microvascular leakage, decreased microvascular perfusion, fibrosis, and HF (58–60).

Earlier we showed that NOD.Cg-Prkdc^scid^Il2rg^tm1Wjl^/SzJ mice reconstituted with human CD34+ hematopoietic stem cells obtained from umbilical cord blood (Hu-mice) can be productively infected with the HIV-1 virus (61–63). These Hu-mice also develop a progressive HF with microvascular leakage and ischemia, akin to that reported in PLWH (64–67), suggesting commonalities in the pathogenesis of HF in Hu-mice and PLWH. Herein, we investigated if the microvascular leakage, fibrosis, ischemia, and HF seen in HIV-infected Hu-mice could be arising from accumulation of MG. Plasma from PLWH and cardiac tissues from deceased HIV-seropositive individuals with HF were also used to confirm elevation in MG.

## Results

### General Characteristics of Hu-Mice

The general characteristics of the animals used in this study are shown in Table 1. Intraperitoneal injection of HIV-1 into Hu-mice led to productive infection; the plasma HIV viral loads four weeks post-infection was 1.5–1.64 × 10^6^ RNA copies/mL and remained elevated during the 16-week period. The percentage of CD4+ T cells in blood declined in HIV-1 infected Hu-mice from 76.6 ± 0.4% to 55.2 ± 2.1% during the course of the study (Supplementary Figure S1A). CD4+ T cells in blood of HIV-infected Hu-mice treated with AAV2/9-Endo Glo1 also declined to 50.2 ± 3.1%. CD8+ T cells increased in blood of HIV-1 infected Hu-mice and HIV-infected Hu-mice treated with AAV2/9-Endo-Glo1 after 16 weeks of infection (Supplementary Figure S1B). The gating strategy used was CD45 → CD3 → CD4/CD8, and the total CD45 and CD3+ T cells did not notably change during the study (Supplementary Figures S1C,D). None of the animals used for this study had to be sacrificed prematurely due to weight loss or graft-vs. host disease.

**Table 1:** General characteristics of animals used in the study.

**Parameter**	**Uninfected Hu-NSG mice (*n* = 6)**	**HIV-infected Hu-NSG mice (*n* = 6)**	**HIV-infected Hu-NSG Treated with AAV2/9-Endo-Glo1 (*n* = 6)**
Duration of infection (weeks)	NA	16	16
Body weight (g)			
4-Weeks	18.3 ± 0.4	18.4 ± 0.3	18.3 ± 0.3
16-Weeks	19.2 ± 0.4	19.0 ± 0.3	19.3 ± 0.5
Plasma HIV viral load (RNA copies/mL)			
4-Weeks	NA	1.6 ± 0.1 × 10^5^	1.5 ± 0.1 × 10^5^
16-Weeks	NA	1.5 ± 0.1 × 10^5^	1.6 ± 0.3 × 10^5^
Plasma SSAO activity (units/mL/2 h)			
4-Weeks	3.1 ± 0.1	4.3 ± 0.1^*^	3.2 ± 0.1^**^
16-Weeks	3.3 ± 0.4	6.1 ± 0.3^*^	4.2 ± 0.3^**^
Plasma HSA-MG eq (μg/ml)			
4-Weeks	30.1 ± 8.1	60.1 ± 10.1^*^	37.2 ± 10.2^**^
16-Weeks	35.2 ± 7.1	88.5 ± 12.3^*^	40.5 ± 9.2^**^

*NA, not applicable*.

* *significantly different from uninfected controls (p < 0.05)*.

** *Significantly different from HIV-1-infected (p < 0.05)*.

### AAV2/9-Endo-Glo1 Treatment Blunt Lefted Ventricular Function in HIV-1 Infected Hu-Mice

**Figure 1:**
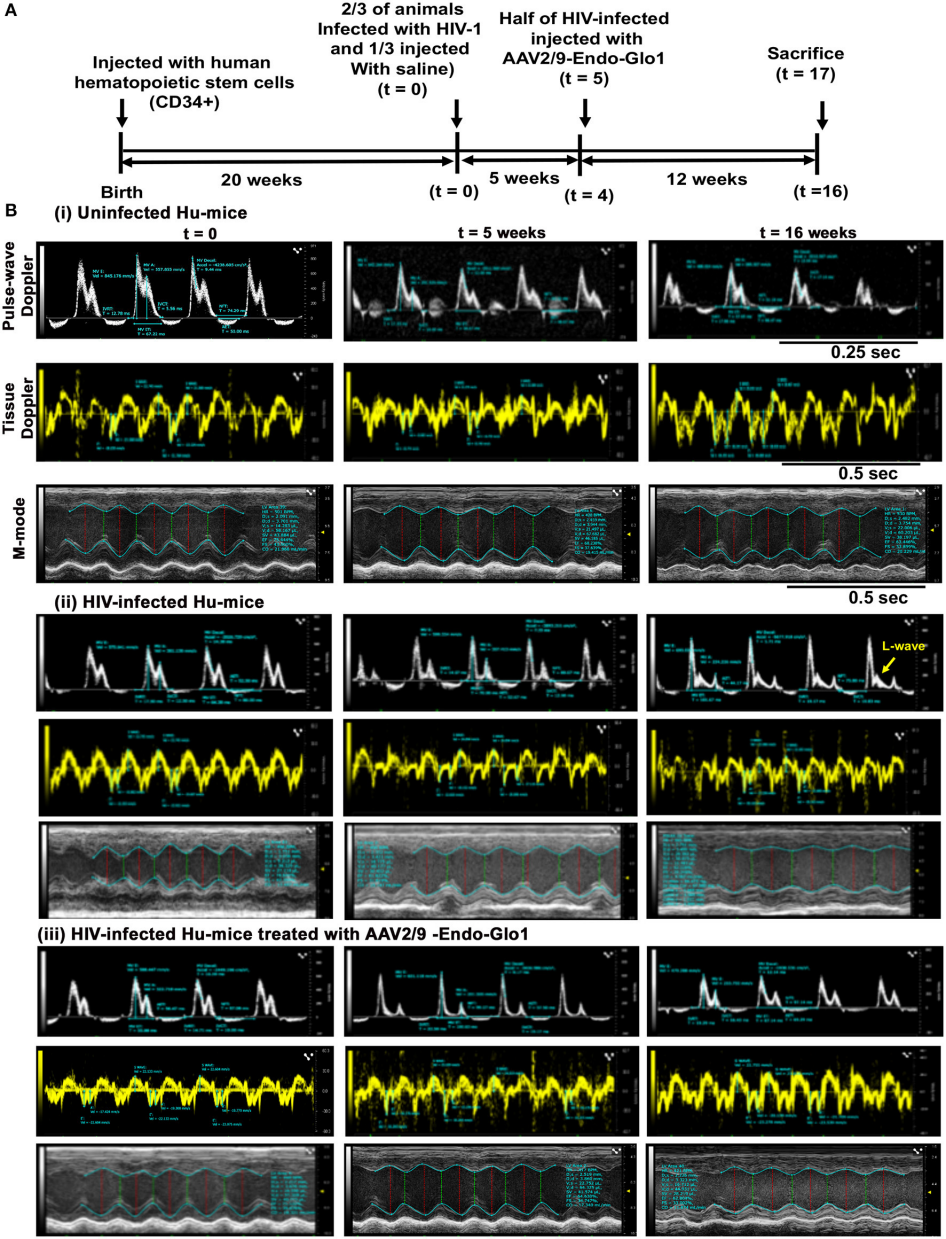
Administration of AAV2/9-Endo-Glo1 shortly after HIV-1 infection attenuated the development of heart failure in humanized mice. **(A)** Experimental scheme for human CD34+ HSC reconstitution, HIV-1 infection, ECHO analysis, Endo-Glo-1 administration, immune cell profiling, and histological evaluations are shown. **(B)** Raw echocardiographic data obtained using Pulse-wave, M-Mode, tissue Doppler in (i) uninfected, (ii) HIV-infected (iii) HIV-infected and treated with AAV2/9-Endo-Glo1. Administration of a single injection of AAV2/9-Endo-Glo1 to hu-mice 5-weeks after HIV-infection attenuated the development of both diastolic and systolic dysfunction as shown at study end. Administration of AAV2/9-Endo-Glo1 also blunted the development of mitral regurgitation (seen as L-wave, yellow arrow).

**Figure 2:**
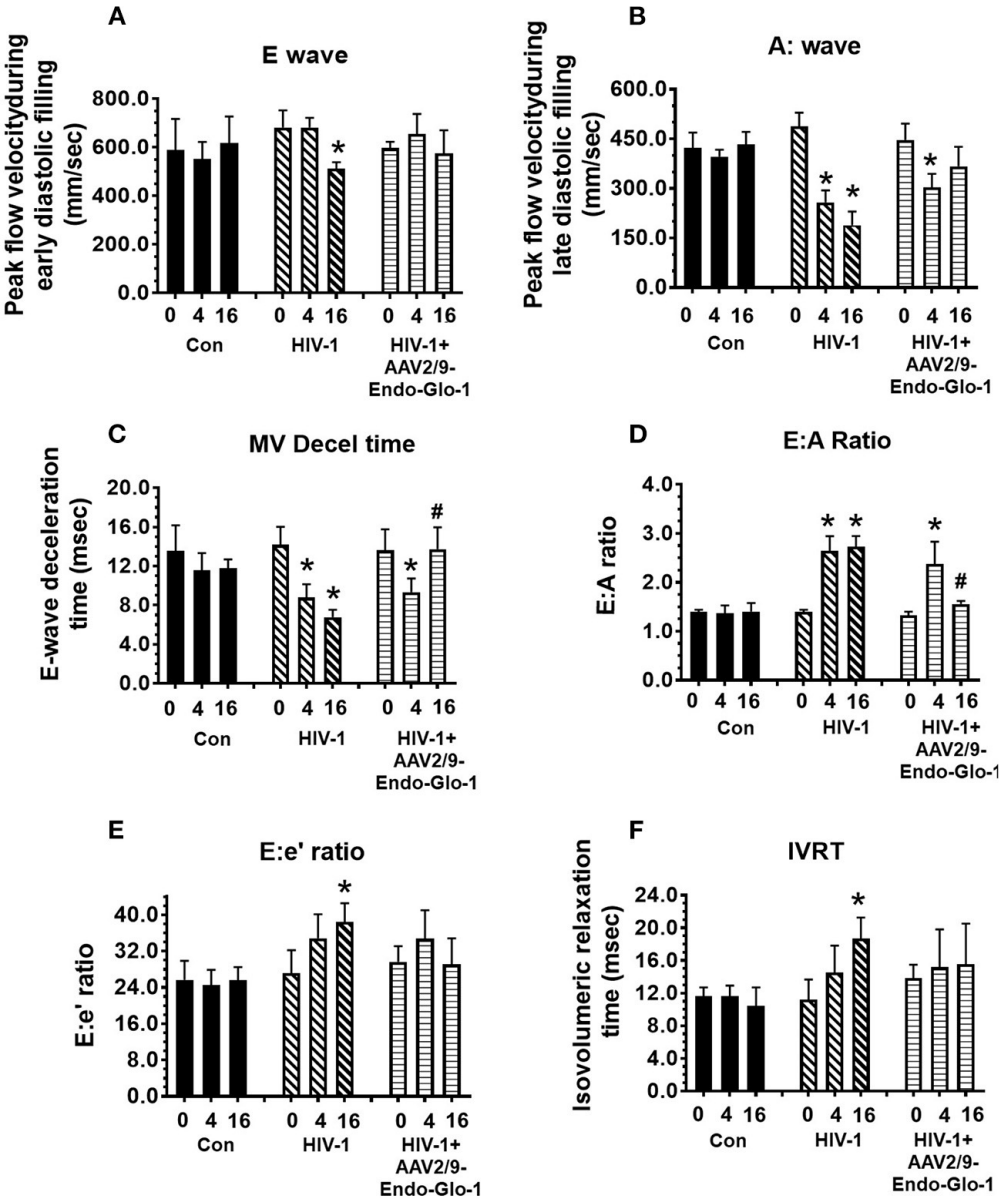
Longitudinal pulse-wave and tissue doppler echocardiography before and after administration of AAV2/9-Endo-Glo1 to Hu-mice after HIV-1 infection reversed/attenuated the development of diastolic dysfunction. The panels below show **(A)** E:wave velocity, **(B)** A:wave velocity, **(C)** E:A ratio, **(D)** E:wave deceleration time, **(E)** E:e ratio and **(F)** isovolumetric relaxation time (IVRT) in uninfected control animals (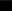) before (0), 5 weeks after and sixteen weeks after injection of saline; in HIV-infected mice (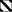) before, 5 weeks and sixteen weeks after HIV-1 infection, and in AAV2/9-Endo-Glo1-treated HIV-infected Hu-mice (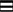) before, 5 weeks after infection with HIV-1, and eleven weeks after administration of AAV2/9-Endo Glo1. Data shown on graphs are mean ± SEM from *n* ≥ 6 mice per group. ^*^Denotes significantly different from uninfected Hu-NSG mice (*p* < 0.05). ^#^denotes significantly different from HIV-1 infected Hu-mice (*p* < 0.05).

**Figure 3:**
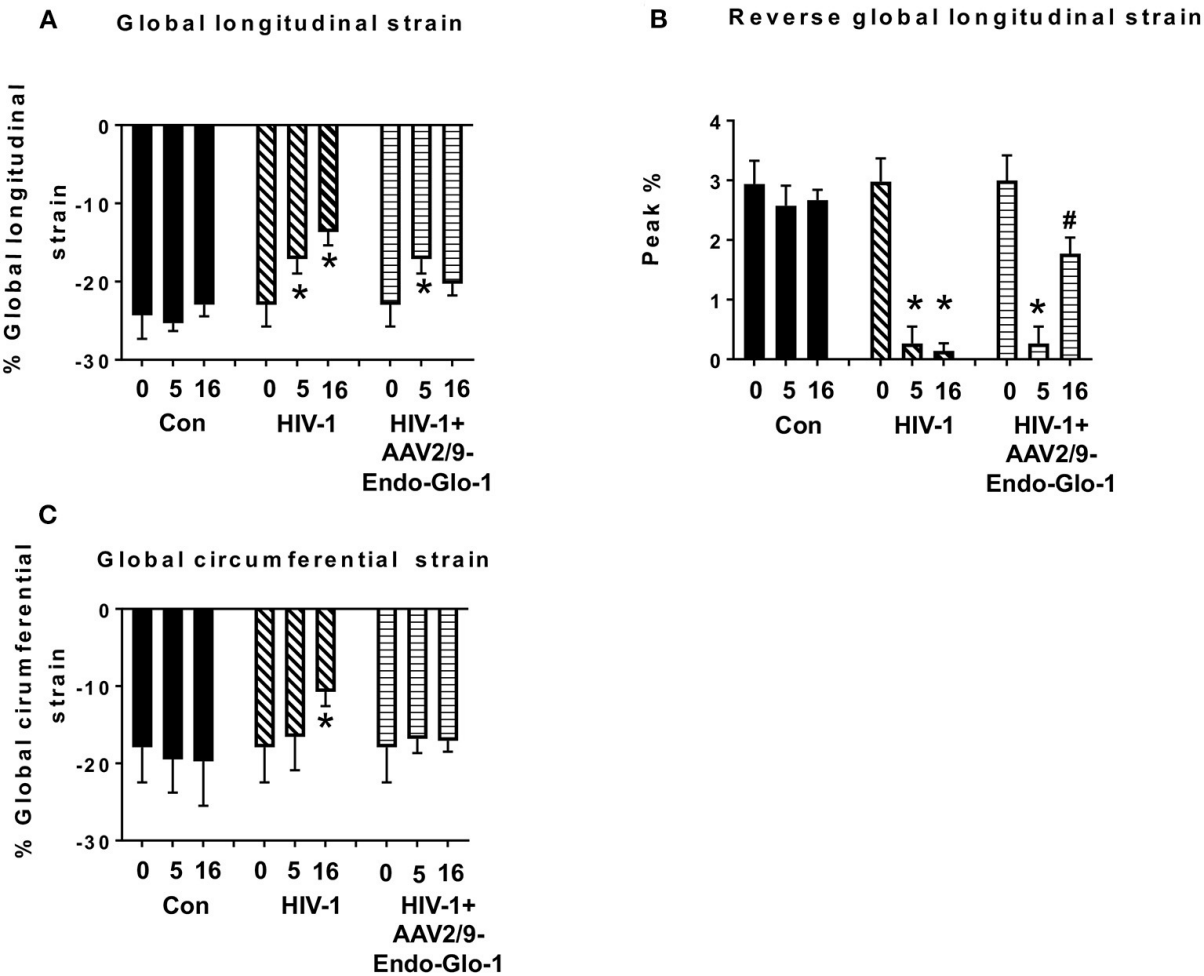
Speckle tracking analysis revealed that administration of AAV2/9-End-Glo1 to Hu-mice after HIV-1 infection attenuated/blunted myocardial strain. **(A)** Global longitudinal strain, **(B)** Reversed global longitudinal strain and **(C)** global circumferential strain in uninfected control animals before injection, 5 weeks, and sixteen weeks after injection of saline; in HIV-infected mice prior to infection with HIV-1, 5 weeks and sixteen weeks after HIV-1 infection, and in AAV2/9-Endo-Glo1-treated HIV-infected Hu-mice before infection, 5 weeks after infection with HIV-1, and eleven weeks after administration of AAV2/9-Endo Glo1 (16 WPI). Data in graphs are mean ± S.E.M from *n* ≥ 6 mice per group female mice per group. ^*^Denotes significantly different from uninfected Hu-mice (*p* < 0.05). ^#^Denotes significantly different from HIV-1 infected Hu-mice (*p* < 0.05).

**Figure 4:**
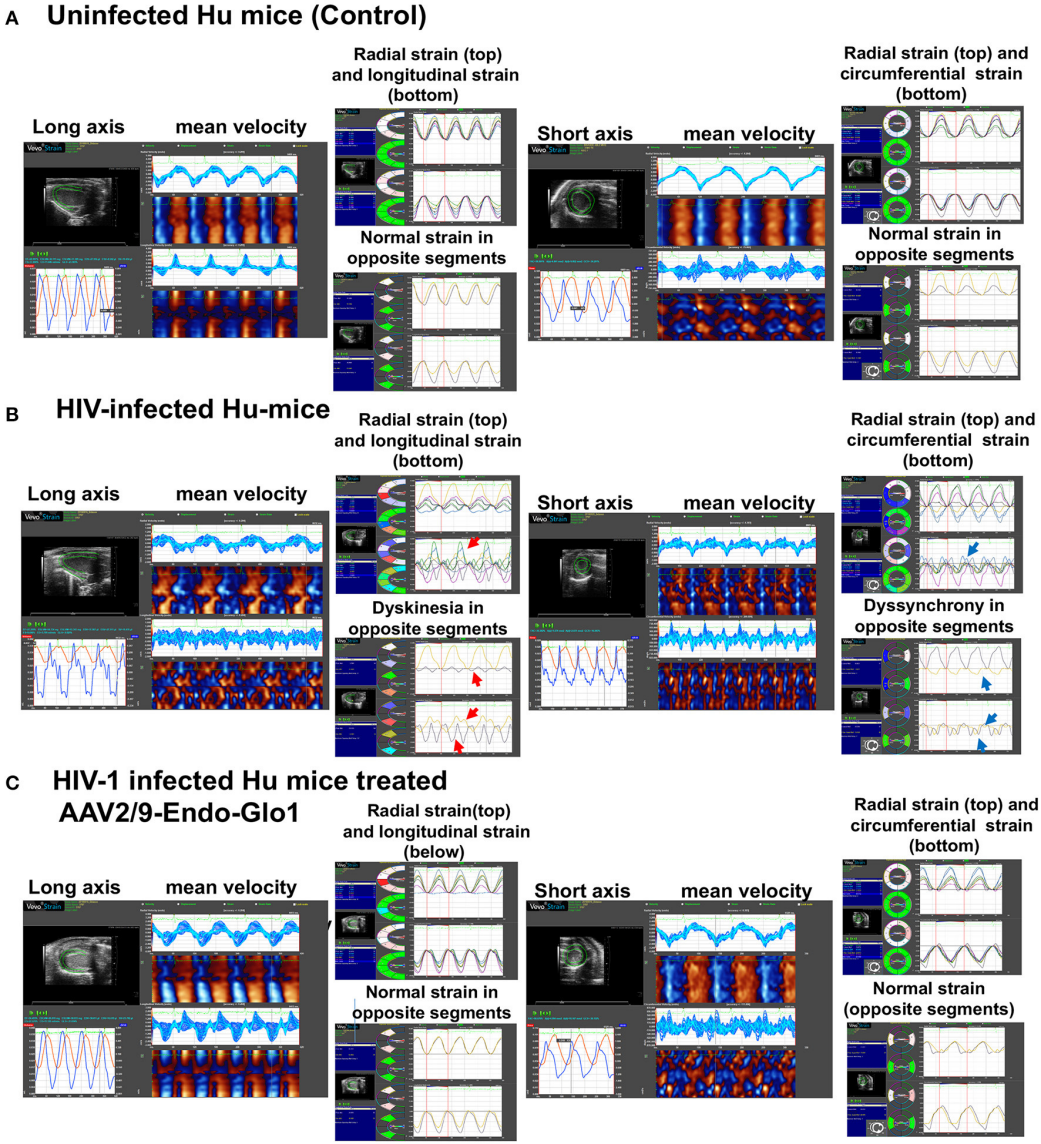
Speckle tracking analysis revealed that administration of AAV2/9-End-Glo1 to Hu-mice after HIV-1 infection attenuated/blunted dyskinesia and dyssynchrony. **(A–C)** Show representative raw data from long (left side) and short axis (right side) velocities during three to four consecutive cardiac cycles (left) in uninfected control animals (*n* = 6) sixteen weeks after injection of saline; HIV-infected hu-mice sixteen weeks after infection (*n* = 7) and HIV-1 infected mice eleven weeks after administration of AAV2/9-Endo Glo1 which is equivalent to 16 weeks post-HIV-infection (*n* = 5). The data shows that 16 weeks after HIV-1 infection, Hu-mice developed dyskinesia [expansion of a wall segment during systole **(B)**, red arrows] and dyssynchrony [opposite walls moving in counter directions **(B)**, blue arrows]. The dyskinesia and dyssynchrony were attenuated in HIV-infected Hu-mice treated with AAV2/9-Endo Glo1.

Non-invasive, multi-modal echocardiography (pulsed-wave, tissue Doppler, M-mode, and speckle tracking) did not reveal any impairments in diastolic or systolic functions of the uninfected humanized mice at the start of the study. At five weeks post-infection, all hu-mice (males and females) developed DD characterized by a reduction in peak late-diastolic transmitral velocity (A-wave), an increase in E:A ratio and an increase in E wave deceleration time [Figure 1B(ii) *t* = 5 weeks and Figures 2B-D]. E:e' ratio and isovolumetric relaxation (IVRT) did not change significantly after five weeks of infection (Figures 2E,F). Other parameters of diastolic functions assayed including, isovolumetric contraction (IVCT), mitral valve ejection time (MV-ET), aortic ejection time (AET), and no flow times (NFT) also did not change after five weeks of infection (data not shown). Consistent with DD, speckle tracking (ST) analyses of B-mode images also revealed a significant lowering in global longitudinal strain (Figure 3A), and a decrease in reverse longitudinal strain (Pk, %) using the reverse peak algorithm (Figure 3B) (63). After five weeks of infection, M-mode Doppler recordings did not show any significant changes in left ventricular systolic functions (percent fractional shortening (FS), percent ejection fraction (EF), left ventricular end diameter–diastole (LVED-diastole), left ventricular end diameter–systole (LVED-systole), and cardiac output (Figure 4).

**Figure 5:**
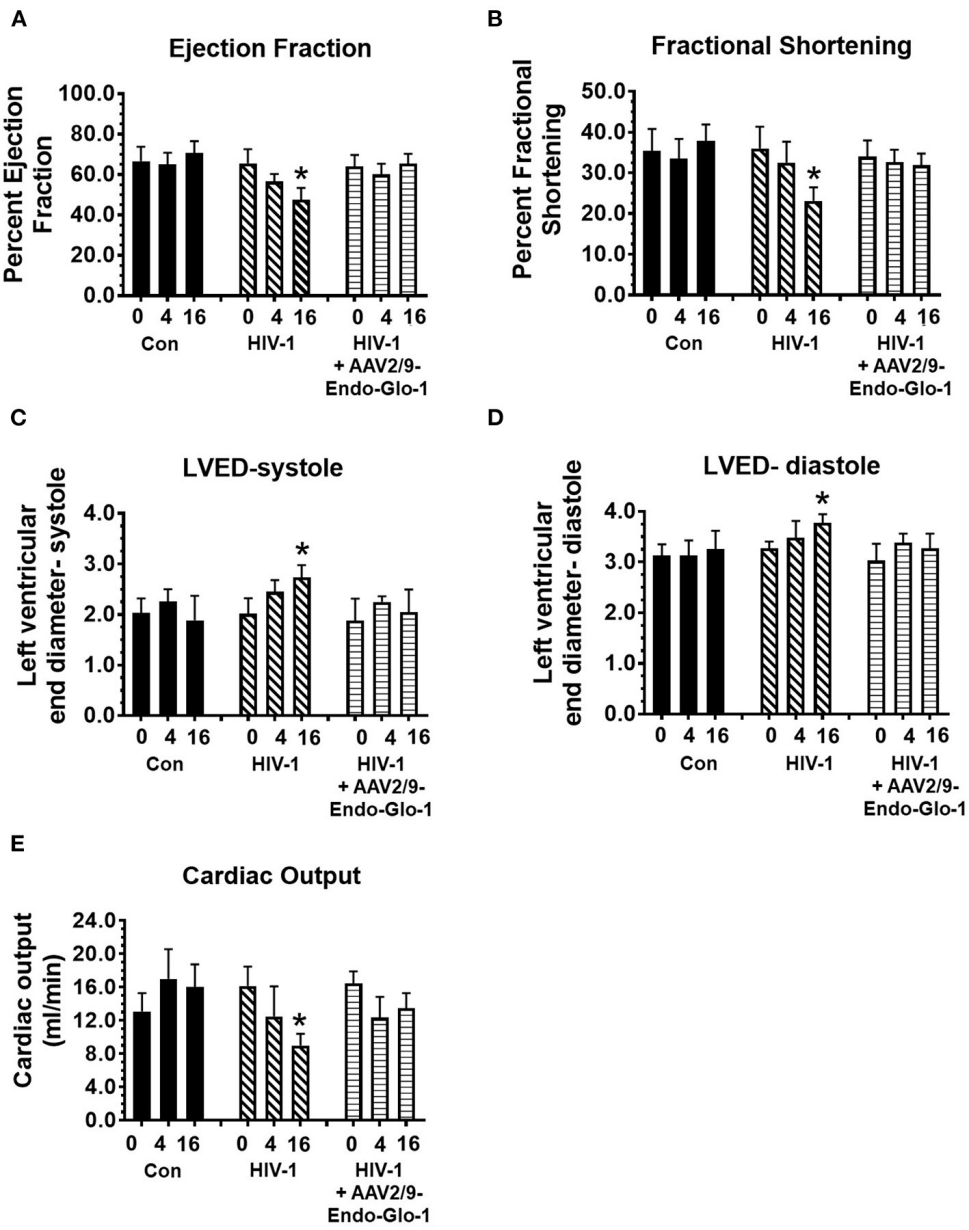
Longitudinal M-Mode echocardiography revealed administration of AAV2/9-Endo-Glo1 to Hu-mice after HIV-1 infection attenuated/blunted systolic dysfunction. **(A)** ejection fraction, **(B)** fractional shortening**, (C)** left ventricular end diameter in systole, **(D)** left ventricular end diameter in diastole, and **(E)** cardiac output in uninfected control animals before injection, 5 weeks and sixteen weeks after injection of saline; in HIV-infected mice before, 5 weeks and sixteen weeks after HIV-1 infection, and in AAV2/9-Endo-Glo1-treated HIV-infected Hu-mice prior to infection with HIV-1, 5 weeks after infection with HIV-1, and eleven weeks after administration of AAV2/9-Endo Glo1. Data in graphs are mean ± S.E.M from *n* ≥ 6 mice per group female mice per group. ^*^Denotes significantly different from uninfected Hu-NSG mice (*p* < 0.05).

After sixteen weeks of infection, pulsed wave and tissue Doppler revealed worsened DD in HIV-1-infected Hu-mice (males and females) with significant declines in E-wave, A-wave and E-wave deceleration time (Figures 1, 2). L-waves were also pronounced in 4/6 HIV-1 infected Hu-mice [Figure 1B(ii), middle right panel, yellow arrow]. E:A ratio, E:e' and IVRT also increased sixteen weeks post-infection (Figure 2) as did global longitudinal strain and global circumferential strain (Figures 3A,C). Reverse global longitudinal strain decreased further (Figure 3B). Strain analyses of long and short axes B-mode images during systole revealed dyskinesis (expansion of a wall segment during systole Figure 4 left panels, red arrows) and dyssynchrony (opposite walls moving in counter directions, Figure 4, right panels blue arrows). After 16 weeks of infection, M-mode echocardiography revealed small but significant declines in FS, EF and cardiac output, and an increase in LVED-diastole and LVED-systole (Figure 5).

A single intravenous injection of AAV2/9-Endo-Glo1 to express the Glo1 five weeks after HIV infection, attenuated impairments in myocardial diastolic and systolic dysfunctions that developed 16 weeks post-infection (Figures 2, 3, 5). Administration of AAV2/9-Endo-Glo1 also attenuated the dyskinesia and the dyssynchrony (Figure 4). Video recordings of parasternal long- and short-axis loops showing direction and magnitude of endocardial deformation between uninfected control, HIV-infected Hu-mice and HIV-infected mice treated with AAV2/9-Endo-1, are shown in Supplementary Videos Files, Videos 1–6. The non-specific virus AAV2/9-Endo-eGFP had no effect on diastolic and systolic parameters (data not shown). In this study, uninfected Hu-NSG mice also did not develop diastolic and systolic deficits during the 16-week study period. Animals used in this study also did not show physical signs of graft-vs-host disease including hair loss, hunch back or reduced mobility.

### AAV2/9-Endo-Glo1 Treatment Blunted Plasma Elevation of MG and Semicarbazide-Sensitive Amine Oxidase (SSAO) in HIV-1 Infected Hu-Mice

After five weeks of HIV-1 infection, MG level was 100.0 ± 6.8% higher in plasma of HIV-1 infected Hu-mice compared to uninfected controls. The activity of the non-selective inflammation enzyme SSAO (the soluble form of vascular adhesion protein-1, VAP-1) was also 38.7 ± 2.3% higher in plasma of HIV-1 infected Hu-mice compared to uninfected controls. After 16 weeks of infection, plasma MG increased further to 250.0 ± 10.3% and SSAO activity increased 84.2 ± 4.3%. A single intravenous injection of AAV2/9-Endo-Glo1 five weeks after HIV-1 infection, attenuated the increases in plasma MG and SSAO as observed at 16 weeks of infection with minimal change in HIV-1 viremia. Uninfected Hu mice showed minimal change in plasma MG and SSAO activity during the study period (Table 1).

### AAV2/9-Endo-Glo1 Treatment Blunted Leakage of Microvessels and Restored Vascular Perfusion in HIV-1 Infected Hu-Mice

**Figure 6:**
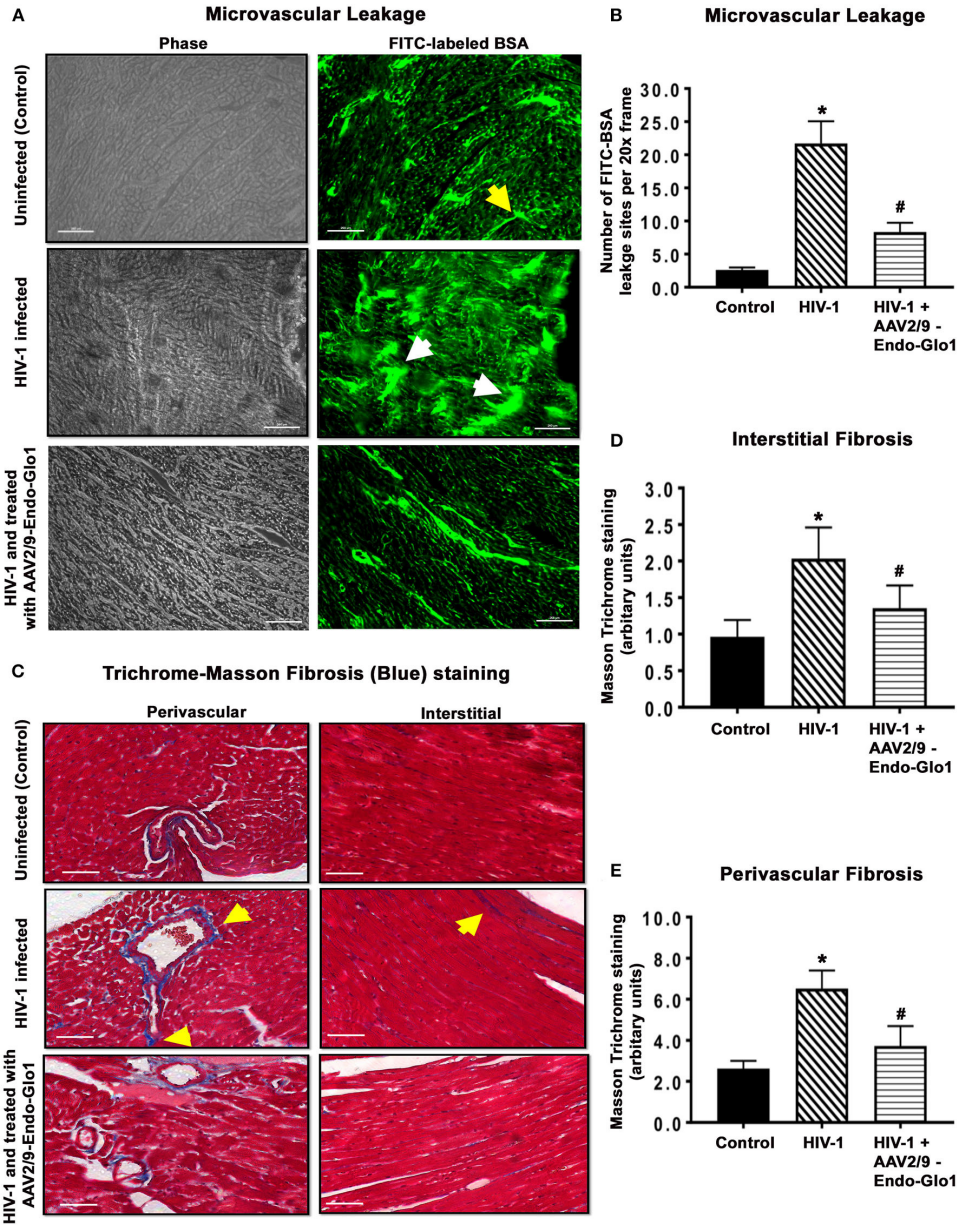
Immunofluorescence and Trichrome-Masson staining revealed administration of AAV2/9-End-Glo1 to Hu-mice shortly after HIV-1 infection attenuated coronary microvascular leakage and fibrosis. **(A)** Representative FITC-BSA images from ventricular sections from uninfected, HIV-infected and AAV2/9-Endo Glo1-treated HIV-infected hu-mice. Yellow arrow points to a perfuse microvessel. White arrows point to microvascular leakage. Graph on right **(B)** shows the relative density of microvessels (<25 μm) perfused with BSA-FITC per 20-x frame. Data in graphs are mean ± SEM from *n* > 20 sections from *n* ≥ 3 mice per group. **(C)** Representative Trichrome-Masson staining for fibrosis in left ventricular sections (interstitial and perivascular) from uninfected, HIV-infected and AAV2/9-Endo-Glo1-treated HIV-infected hu-mice at seventeen-weeks study protocol. Graphs on right **(D,E)** are mean ± S.E.M from *n* ≥ 3 mice per group. ^*^Denote significantly different (*p* < 0.05) compared to saline injected humanized mice. ^#^Denotes significantly different from HIV-1 infected Hu-mice (*p* < 0.05). White scale bar indicates 200 μm.

In uninfected control animals, the green fluorescence of injected FITC-labeled BSA (FITC-BSA) appeared throughout the vascular network of mice, indicative of perfusion of the coronary microvessels. Larger diameter vessels which typically contain more blood had more FITC-BSA fluorescence compared to the smaller capillaries (Figure 6A, yellow arrow). There was also minimal leakage of the FITC-BSA from the vasculature into the myocardium of uninfected mice. Interestingly, in ventricular tissues in HIV-1 infected animals, FITC-BSA fluorescent was present as “blobs” in some regions, indicative of leakage from the confines of the microvessels into the myocardium (Figure 6A, white arrows). Intravenous administration of AAV2/9-Endo-Glo1 to Hu-mice five weeks after HIV-1 infection blunted the impairment in microvascular permeability and microvascular leakage seen in heart at seventeen weeks post-infection (Figure 6A, lower right panel). Quantitation of microvascular leakage is shown in Figure 6B.

Myocardial tissues from hu-mice seventeen weeks following viral infection contained extensive perivascular and interstitial fibrosis. This was indicated by blue Masson's Trichrome staining (Figure 6C, middle panels, blue staining, yellow arrows), as compared to uninfected controls which exhibited minimal Masson's Trichrome blue staining (Figure 6C, upper left panel). Administration of AAV2/9-Endo-Glo1 five weeks after HIV-1 infection blunted the increase in interstitial and perivascular fibrosis seen after 17 weeks post-infection (Figure 6C). Quantitation of data are shown in Figures 6D,E.

**Figure 7:**
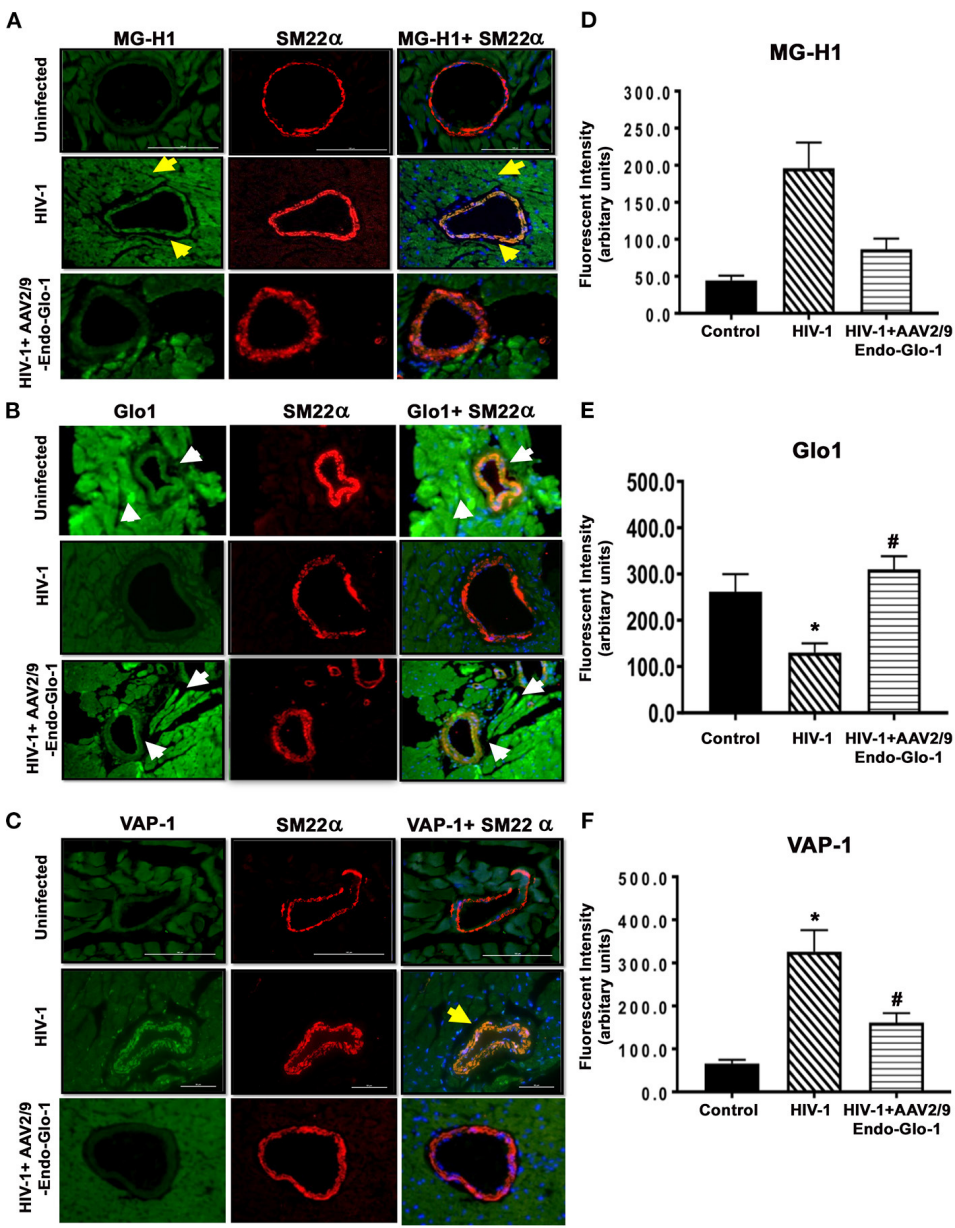
Immunofluorescence assays revealed increasing expression of Glo1 in HIV-infected Hu-mice using AAV2/9-Endo-Glo1 attenuated MG-H1 adduct and the inflammation-induced protein VAP-1. **(A–C)** Shows representative MG-H1, Glo1, and VAP1 immunofluorescence staining in ventricular sections from uninfected, HIV-infected and AAV2/9-Endo Glo1-treated HIV-infected Hu-mice. Yellow arrows in **(A)** shows high immuno-fluorescence for MG-H1 in ventricular tissues from uninfected control tissues. White arrows in **(B)** shows high levels of Glo1 in ventricular shows from uninfected control and AAV2/9-Endo Glo1-treated HIV-infected Hu-mice. Yellow arrow in **(B)** shows high immuno-fluorescence for VAP-1 in ventricular tissues HIV-infected Hu-mice. Graph on right **(D–F)** are mean ± S.E.M for *n* ≥15 sections obtained from minimum of (*n* ≥ 3) animals per group. ^*^Denote significantly different (*p* < 0.05) compared to saline control. ^#^Denotes significantly different from HIV-1 infected Hu-mice (*p* < 0.05).

After seventeen weeks of infection, the MG adduct (MG-hydroxyimidazole, isomer 1, MG-H1) was also increased by 400% in ventricular tissues [Figure 7(i), middle panels, yellow arrow]. Glo1 immunofluorescence was also reduced by 40%, compared to uninfected controls [Figure 7(ii), middles panels]. VAP-1 was upregulated in vascular smooth muscle cells [Figure 7(iii), middle panels]. Administration of AAV2/-Endo-Glo1 five weeks post-HIV-1 infection blunted the upregulation of MG-H1, VAP-1 and the loss of Glo1 seen in hearts of Hu-mice, seventeen weeks post-HIV-1 infection (Figure 7, right panels).

### AAV2/9-Endo-Glo1 Treatment Blunted Endothelial Cell Dysfunction in HIV-1 Infected Hu-Mice

**Figure 8:**
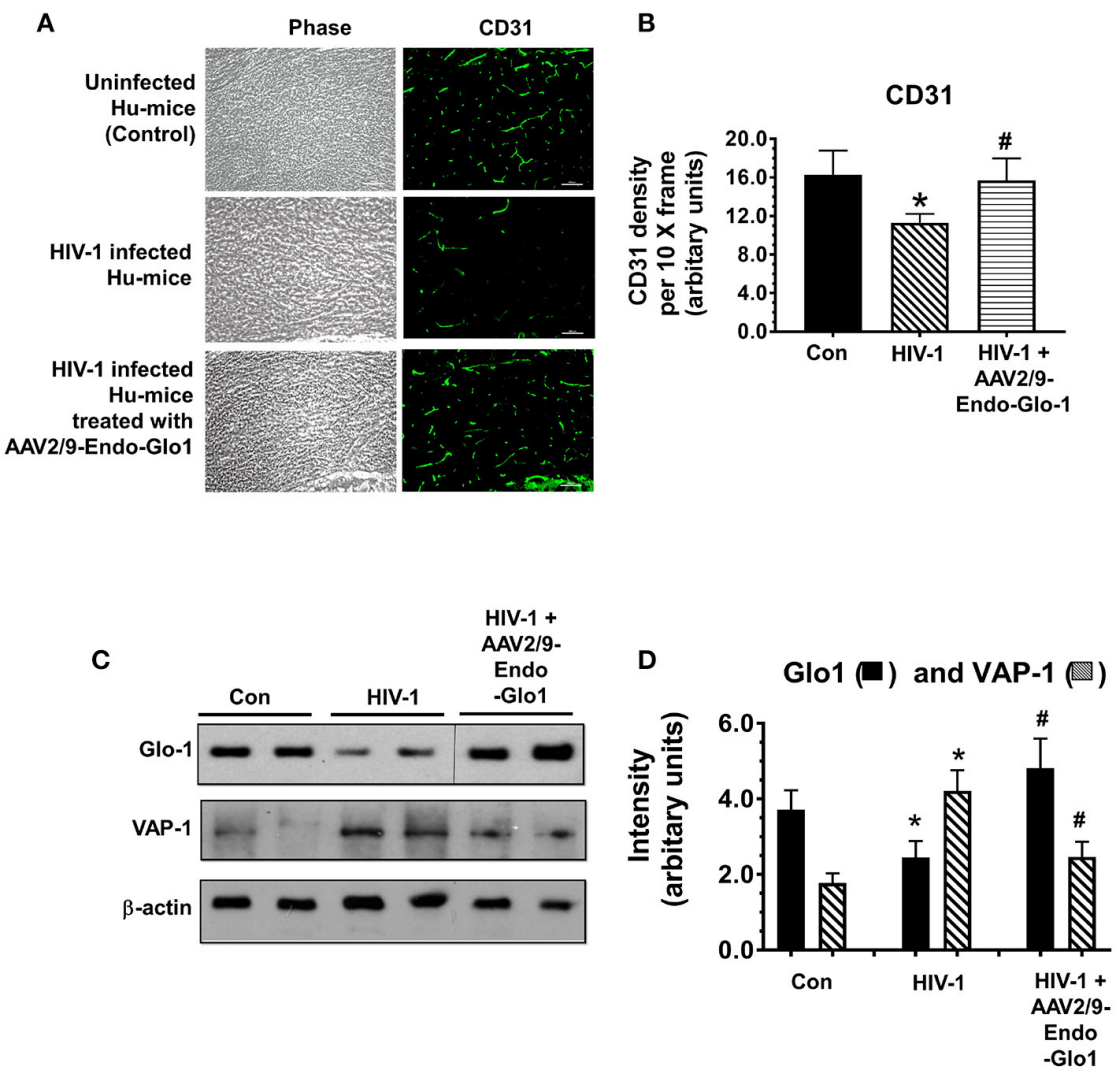
Increasing expression of Glo1 in hearts of HIV-infected Hu-mice using AAV2/9-Endo-Glo1 attenuate loss of CD31 and decrease in VAP-1. **(A)** Immuno-fluorescence analysis to look for the endothelial cell marker CD31 in ventricular tissues from uninfected, HIV-infected, and AAV2/9-Endo Glo1-treated HIV-infected hu-mice. Graph on right **(B)** shows mean ± S.E.M relative intensity from *n* > 15 sections from *n* ≥ 3 mice per group, per 10-x frame. **(C)** Representative autoradiograms for Glo1 and VAP1 in ventricular homogenates from uninfected, HIV-infected and AAV2/9-Endo Glo1-treated HIV-infected Hu-mice. Graph on right **(D)** are mean ± S.E.M for ventricular homogenates from (*n* ≥ 3) animals per group and done in duplicates. ^*^Denote significantly different from control (*p* < 0.05), ^#^Denote significantly different from T1DM (*p* < 0.05). Scale bar at bottom of each image = 50 μm.

Immunofluorescence staining was also conducted for the endothelial cell marker CD31, to assess the integrity of endothelial cells (ECs) in the coronary microvasculature. After seventeen weeks of infection, CD31 immunofluorescence was reduced by 27.8 ± 2.1% in ventricular sections from HIV-infected animals compared to uninfected controls (Figure 8A, and graphs on right, and Figure 8B). Administration of AAV2/-Endo-Glo1 blunted the upregulation of the loss of CD31 seen in the myocardium of Hu-mice 16 weeks post-HIV-1 infection (Figure 8A).

### AAV2/9-Endo-Glo1 Treatment Restored Glo1 and Decreased VAP-1 Protein in Ventricular Homogenates From HIV-Infected Hu-Mice

Western blot analyses conducted to validate changes in ventricular levels of Glo1 and VAP-1 seen in immunofluorescence assays. In this study we found that after seventeen weeks of HIV-1 infection, cardiac Glo1 level was 40.2 ± 3.5% lower than that in control animals (Figure 8C). Myocardial VAP-1 level also increased by 58.6 ± 6.5%. A single intravenous injection of AAV2/9-Endo-Glo1 to hu-mice five weeks after HIV-1 infection, increased myocardial Glo1 protein and attenuated the increase in VAP-1 as observed at seventeen weeks following viral infection (Figure 8D).

### Increased MG and SSAO in PLWH Plasma and Increased MG, Glo1, and VAP-1 in Autopsied Cardiac Tissues in HIV+ Individuals

**Figure 9:**
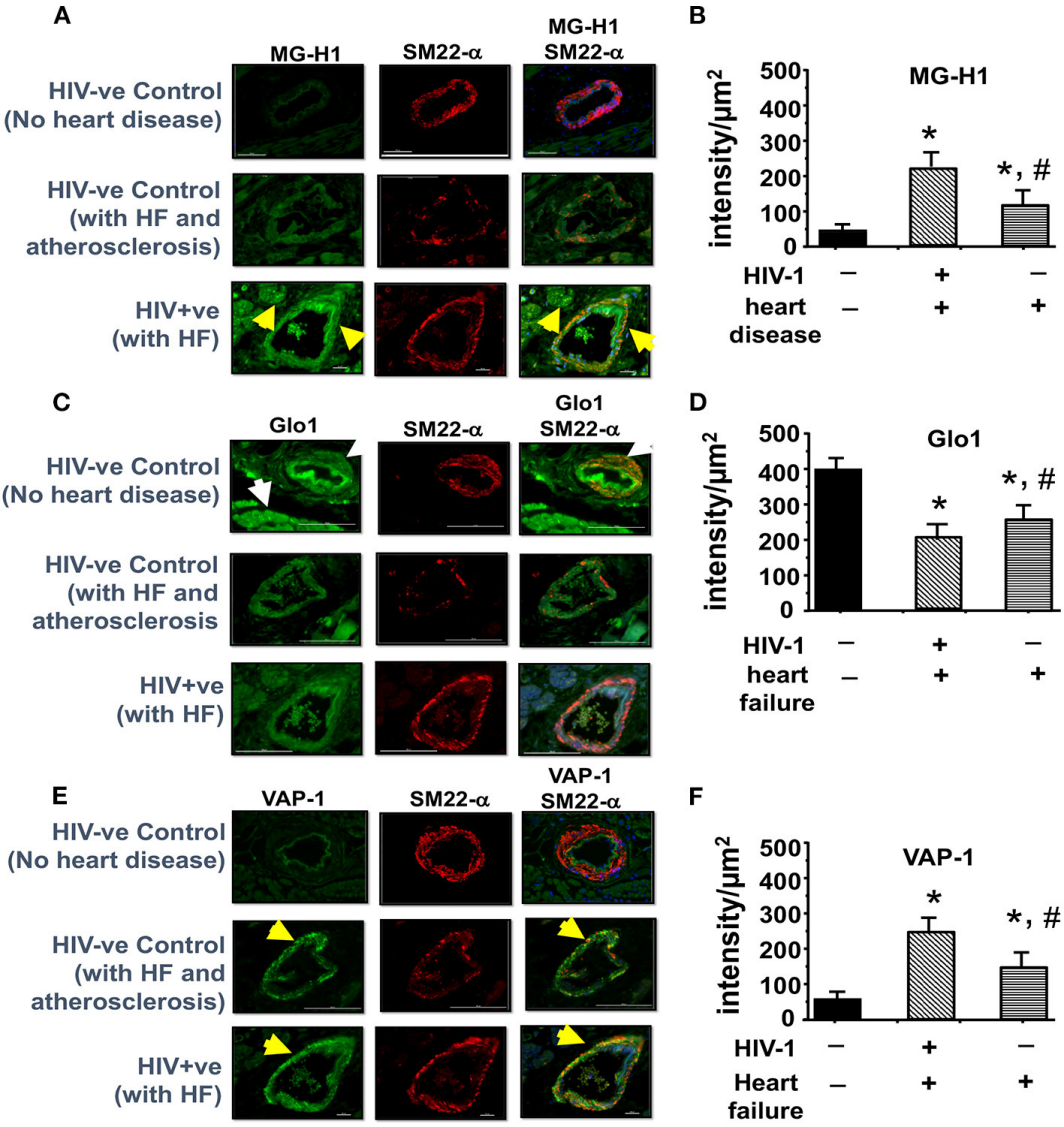
Immunofluorescence revealed increasing expression of MG-H1 and VAP-1 and decreased Glo1 in autopsied left ventricular tissues from decreased HIV+ patients. **(A,C,E)** shows representative MG-H1, Glo1, and VAP1 immunofluorescence staining in autopsied ventricular sections from deceased HIV- patients without heart failure, uninfected controls with atherosclerosis, and HIV+ patients with heart failure. Yellow arrows in **(A,C)** show elevated MG-H1 and VAP-1 in autopsied ventricular sections from non-HIV-infected controls with atherosclerosis and HIV+. White arrows in **(B)** shows elevated Glo1 in autopsied ventricular sections from deceased uninfected patients without heart failure. Graph on right **(B,D,F)** are mean ± S.E.M for (*n* ≥ 6) sections obtained from *n* = 7 patients per group. ^*^Denote significantly different (*p* < 0.05) compared to uninfected control without HF. ^#^Denotes significantly different from HIV+ (*p* < 0.05).

The amount of MG and SSAO activity in plasma of PLWH, and MG-H1, Glo1 and VAP-1 in autopsied cardiac tissues from deceased HIV-1 sero-positive individuals with HF were assessed to validate the clinical significance of our pre-clinical findings. In plasma of PLWH (age 48.9 ± 3.5 years, duration of infection, 8.55 ± 2.12 years, plasma viral load <20 RNA copies/ml, Supplementary Excel File, patient plasma), MG was 3.2-fold higher when compared to uninfected controls (1607.2 ± 89.3 HSA-MG eq (μg/mL) compared to 502.6 ± 61.8 HSA-MG eq (μg/mL)). Mean SSAO activity was also 60% higher compared to uninfected controls (15.5 ± 1.6 units/mL/2 vs. 5.0 ± 0.5 units/mL/2 h, Supplementary Excel File, patient plasma). In autopsied ventricular tissues from HIV+ patients with HF MG-H1 was 4.2 ± 0.3-fold higher in vascular smooth muscle cells than control HIV-1 seronegative samples with no HF and 2.0 ± 0.2-fold higher than HIV- patients with atherosclerosis and HF (Figure 9, upper panels and graph bottom left, also see Supplementary Excel File, autopsied tissues). Glo1 levels were about 50% lower in cardiac tissues from HIV+ with HF as compared to HIV- with no HF and 35 ± 5.1% lower than HIV- group with atherosclerosis and HF (Figure 9, middle panels and middle graph). VAP-1 was 5.0 ± 0.3-fold higher in HIV-1 seropositive group with HF as compared to HIV-1 seronegative with no HF and 3.1 ± 0.2-fold higher in HIV-1 seronegative patients with atherosclerosis and HF.

## Discussion

HF remains a major cause of morbidity and mortality in >40% of PLWH (4–13). Drugs to blunt or slow the progression of HF in PLWH are not available in part to an incomplete understanding of its underlying cause(s). Herein, we demonstrate for the first time that elevation of the cytotoxic glycolysis byproduct MG is an initiating cause for HF during HIV-1 infection. This conclusion is based on new findings obtained from infected Hu-mice, plasma from PLWH and autopsied cardiac tissues from deceased HIV-1 seropositive individuals with HF. MG has been previously linked to HF in other inflammatory diseases (60, 68).

In this study we confirmed our HIV-infected Hu-mice developed a progressive HF using longitudinal echocardiography and histopathological analyses (63). At five weeks post-infection, HIV-infected Hu-mice developed diastolic dysfunction with/without mitral regurgitation that progressed to systolic dysfunction as the duration of infection increased (63). We also found for the first time a progressive increase in plasma MG with worsening HF. Prior studies have reported that following HIV-1 infection, immunocytes upregulate glucose transporter 1 (GLUT1) and increase aerobic glycolysis to generate the necessary substrates needed for HIV-1 replication (43–46). Since 0.1% of glucotriose flux is converted to MG (49, 50), an increase in glycolysis in immunocytes could account in part for the increase in MG. However, additional work is needed to confirm this. Accumulation of MG in plasma and cardiac tissues could also arise from a reduction in its degradation. Using immunofluorescence and Western blot assays, in this study we found for the first time a reduction in Glo1 protein in hearts of Hu-mice seventeen weeks post-infection. These data indicate that the accumulation of MG during HIV-1 infection is also arising in part from a reduction in its degradation.

Next, we assessed if increasing expression of Glo1 would blunt accumulation of MG in HIV-infected hu-mice. Under non-stressed conditions, the nuclear factor erythroid 2-related factor 2 (Nrf2) binds to the antioxidant response element (ARE) on the promoter region of the Glo1 gene induces expression of Glo1 (54, 69). However, under inflammatory as is the case in HIV-1 infection, activated NF-κB would compete with Nrf2 to suppress Glo1 expression (49, 56, 57). As such, to induce expression of Glo1 under inflammatory conditions, we replaced the endogenous CMV promoter of AAV2/9 with the promoter of inflammation-induced protein endothelin-1 (58, 60). Using this strategy, we show for the first time that increasing expression of Glo1 in hearts of HIV-infected Hu-mice decreased MG and blunted the HF seen 16 weeks post-infection, establishing that elevated MG is an underlying cause of HF in HIV-1-infected Hu-mice. It should be mentioned that in this study AAV2/9-Endo-Glo1 was not given to uninfected Hu-mice as these mice have low systemic and tissue inflammation, and as such this viral construct would not express Glo1 (58, 60).

Studies were then conducted to delineate mechanisms by which elevated MG elicited HF during HIV-1 infection. First, we found that increasing Glo1 expression blunted the loss of CD31, and microvascular leakage seen in HIV-1 infected Hu-mice, indicating that Glo1 was protected vascular endothelial cells (ECs) in the heart from MG insults. Earlier we showed that microvascular ECs are especially susceptible to elevation in plasma MG due to their low expression of Glo1 (58). We also showed that chronic exposure of microvascular ECs to MG decreased expression of their tight junction proteins (58). Second, we found that increasing Glo1 in the heart of HIV-1-infected hu-mice attenuated perivascular and interstitial fibrosis (60) indicating that the myocardial fibrosis that developed in hearts of HIV-infected Hu-mice were linked to elevated MG. Although the specific molecular pathways by which elevated MG induce fibrosis is not clear, we posit that MG-induced reduction in tight junction proteins would increase extravasation of blood substances and immunocytes into the cardiac interstitium, triggering inflammation, activating matrix metalloproteinases and the deposition of collagen fibers (70). Third, in this study we found that increasing Glo1 expression in hearts of HIV-1 infected mice blunted plasma SSAO and cardiac VAP-1 expression, consistent with the notion that elevation in MG is contributing to systemic and cardiac inflammation (71–73). However, additional studies are needed to determine the cause-effect relationships between MG, cardiac inflammation, NF-κB and NRLP3 inflammasome activation (52, 53).

Plasma from PLWH and autopsied cardiac tissues were assayed for MG, Glo1, SSAO, and VAP-1 to define the clinical relevance of our findings. In plasma from PLWH with low HIV-viremia and autopsied tissues from deceased HIV-1 seropositive individuals, MG levels were 3.7-fold and 4.2-fold higher than that in uninfected controls, respectively. SSAO was also 60% higher in plasma of PLWH compared to uninfected controls. In autopsied cardiac tissues from deceased HIV+ individuals, VAP-1 was 5.0-fold higher and Glo1 was 50% lower as compared to uninfected controls without HF. Thus, the elevation in MG and the reduction in Glo1 observed in Hu-mice is of clinical relevance to HF for PLWH.

There are some limitations with the present study. First, although our data show that MG (measured as its surrogate MG-H1) is elevated in plasma and cardiac tissues from HIV-infected Hu-mice, PLWH and autopsied cardiac tissues from HIV-seropositive deceased individuals, the underlying cause for this is not well-delineated. In the present study, we focused on expression of Glo1. However, Glo-1 degrades the hemiacetal formed between MG and reduced glutathione, GSH (49, 50). Thus, a reduction in GSH could result in MG accumulation. As such, additional studies will be needed to determine total glutathione, the ratio of reduced and oxidized glutathione, and the activities of the two enzymes involved in the synthesis of GSH, namely γ-glutamylcysteine ligase (ligates l-glutamate and l-cysteine), and glutathione synthetase (adds glycine to γ-glutamylcysteine) in hearts of HIV-infected Hu-mice. Second, ischemic regions were observed in hearts of HIV-1 infected Hu-mice as well as in PLWH (63, 66, 67). Under normoxia, the heterodimeric transcription factor that regulates cellular and systemic adaptive responses hypoxia-inducible factor 1α (HIF-1α) is targeted for degradation by the proteasome via hydroxylation of proline residues, mediated by the oxygen-dependent prolyl hydroxylase domain (PHD) family of enzymes (74, 75). When oxygen delivery is compromised, as is the case with ischemia, the PHD enzymes are inhibited, and HIF-1α escapes hydroxylation, allowing it to migrate to the nucleus and induce transcription of HIF-1 target genes, including those involved in glycolysis and erythropoiesis. HIF-1α also binds to the antioxidant response element (ARE) on the promoter region of the *GLO1* gene to inhibit Glo1 expression (49, 56, 57). Thus, ischemia and activation of HIF-1α will also inadvertently lead to an increase in MG. Additional work will also be needed to investigate if inhibitors of HIF-1α are cardio-protective in the setting of HIV-1 infection. Third, the present study focused on EC dysfunction and microvascular leakage, vascular changes that are known mediators of inflammation, fibrosis, and HF. However, supraphysiologic level of MG can also perturb intracellular Ca^2+^, induce reactive oxygen species (ROS), and form adducts on accessible basic moieties of proteins that could negatively impact the function of cardiac myocytes (59). In the future, we will assess if myocytes from HIV-1 infected Hu-mice have impaired Ca^2+^ homeostasis, increased ROS production, and diminished contractile properties.

**Figure 10:**
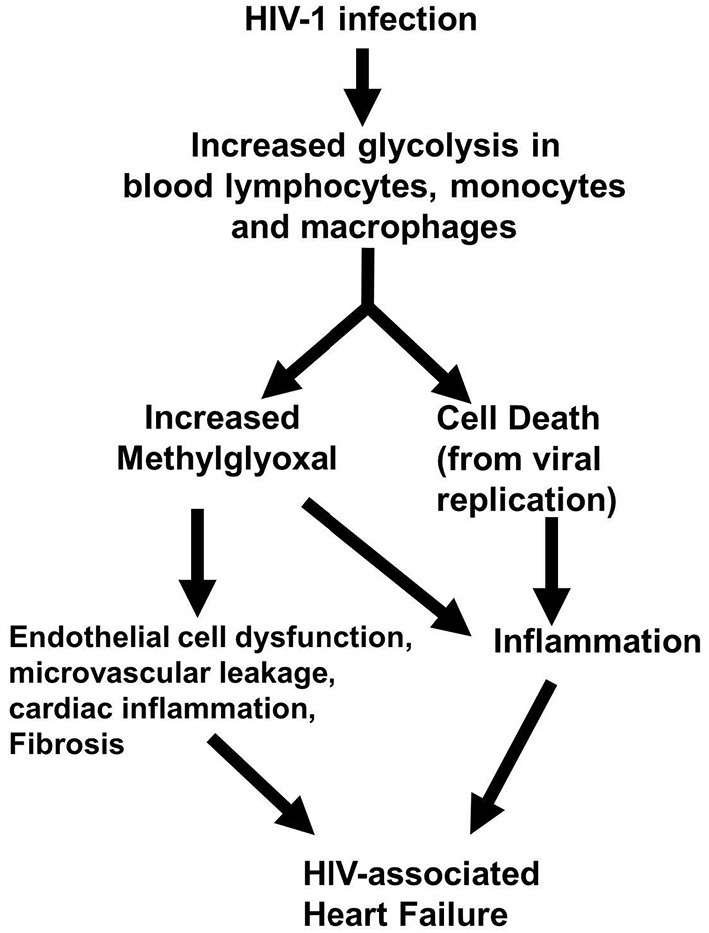
Working hypothesis by which elevation in MG induces HF during HIV-1 infection.

In conclusion, the present study shows for the first time that early-onset HF seen in HIV-1-infected Hu-mice is arising in part from accumulation of the cytotoxic glycolysis metabolite MG. We also showed that this elevation in MG is arising in part from a decrease in its degradation and precipitating dysregulation of coronary microvascular ECs, microvascular leakage, and fibrosis (Figure 10). We posit that an elevation in MG could also be an underlying cause increase in systemic and cardiac inflammation seen during HIV-1 infection by activating NF-κB and NRLP3 (4, 5, 19–21). These data also suggest that therapeutic strategies to lower MG levels may be useful in reducing inflammation and HF during HIV-1 infection.

## Materials and Methods

### Antibodies and Reagents

Human hematopoietic stem cell enrichment was done using magnetic beads conjugated CD34+ antibodies from Miltenyi Biotec Inc. (Auburn, CA, USA). The human immune cell reconstitution in mice was assessed by flow cytometry using fluorescence conjugated primary antibodies to the human antigens CD45, CD3, CD19, CD4, CD8, and CD14 from BD Pharmingen (San Diego, CA, USA). Antibodies for immunohistochemistry were obtained from AbCam Inc., Cambridge MA [anti-VAP-1, rabbit polyclonal, Cat # Ab187202; anti-TAGLN (SM22α), goat polyclonal, Cat # Ab10135]; Hycult Biotech, Wayne PA (mouse monoclonal, MG-H1, Cat # HM5017) and Santa Cruz Biotechnology Inc., Santa Cruz, CA [Glo-I [FL-184]], rabbit polyclonal, Cat # SC-67351; the non-selective inflammation-induced protein VAP-1 [E-19], goat polyclonal, Cat # sc-13741; and actin (1–19), goat polyclonal Cat # SC-1616). Secondary antibodies were obtained from Invitrogen Life Technologies (chicken anti-rabbit IgG coupled to Alexa Fluor 488, Cat # A21441; chicken-anti-mouse IgG coupled to Alexa Fluor 488, Cat # A21200; donkey anti-goat IgG coupled to Alexa 594 Cat # A11058); Santa Cruz Biotechnology, Inc. (Donkey anti-rabbit IgG-HRP, Cat # sc-2305 Donkey anti goat IgG-HRP Cat # sc-2304). Fluorescein isothiocyanate-labeled bovine serum albumin labeled with (FITC-BSA, Cat # A9771), Trichrome-Masson staining kit (Cat # HT15-1KT) and Fluoroshield with DAP1, (Cat # F6057) were from Sigma-Aldrich (St Louis, MO). Semicarbazide-sensitive amine oxidase (soluble form of VAP-1) assay kits (Cat # SSAO100) was obtained from Cell Technology, Inc. (Mountain View, CA). OxiSelect™ MGO competitive ELISA kit (Cat # STA-811) was obtained from Cell BioLabs Inc., San Diego CA. All other reagents were from commercial sources.

### Ethics Statement

All experimental protocols involving the use of laboratory animals were approved by the University of Nebraska Medical Center (UNMC) Institutional Animal Care and Use Committee (IACUC) ensuring the ethical care and use of laboratory animals in experimental research. All animal studies were performed in compliance with UNMC institutional policies and NIH guidelines for laboratory animal housing and care. Human CD34+ Hu-NSG were isolated from umbilical cord blood obtained from UNMC labor and delivery department at UNMC with written consents from adult parents to use the remaining or discarded biological material for research. Samples were collected without identifiers under UNMC Institutional Review Board (IRB) exempt. The UNMC institutional IRB determined that these studies using anonymized cord blood samples doesn't constitute human subject research as defined at 45CFR46.102(f). We regularly collect the cord blood samples and isolate CD34+ hematopoietic cells which are either injected immediately into mice or stored in liquid nitrogen for future human reconstitution. NOD.Cg-PrkdcscidIl2rgtm1Wjl/SzJ, NSG mice were obtained from the Jackson Laboratories (Bar Harbor, Maine, USA; stock number 005557), and a breeding colony was developed at the University of Nebraska Medical Center. All animal procedures are approved under the IACUC protocols 18-110-08 and 10-107-01 for ECHO procedures (61–63).

### Autopsied Ventricular Tissues From Deceased HIV+ Individuals and Plasma From PLWH

De-identified, autopsied left ventricular tissues on glass slides (consecutive sections) from seven de-identified HIV+ patients with myocardial dysfunction (4 males and 3 females) were obtained from the National NeuroAIDS Tissue Consortium (NNTC), under approved protocol # R605. Age, HIV-1 infection duration, plasma viral load and ARDs taken are in Supplementary Excel File-a. Deidentified autopsied cardiac tissues from seven patients with cardiac hypertrophy and atherosclerosis (4 males and 3 females) and from seven uninfected individuals who died in accidents with no reported history of heart diseases were obtained from the tissue bank at the UNMC. De-identified plasma from ten HIV-infected and uninfected “control” patients were also obtained from the UNMC tissue bank. Plasma viral load and ART taken are in Supplementary Excel File-b.

### Construction of Adeno-Associated Virus Containing Glo1

The University of Pennsylvania Vector Core Facility constructed an adeno-associated virus, AAV2/9 containing glyoxalase-1 driven by the promoter of the inflammation-induced protein, endothelin-1 (AAV2/9-Endo-Glo1) with support from the Gene Therapy Resource Program, GTRP # 1053 (58, 60).

### Generation of Humanized Mice

Humanized mice (Hu-mice) were prepared as described in prior publications (61–63). Shortly after birth, NOD.Cg-Prkdc^scid^ Il2rgt^m1Wjl^/SzJ mice were briefly irradiated with a sub-lethal dose of radiation (1Gy) using a RS-2000 X-Ray Irradiator (Rad Source Technologies). CD34+ cells (50,000 cells/mouse) enriched from human cord blood (>90%) were then injected intra-hepatically and left for humanization. At monthly intervals mice were bled via a submandibular vein into EDTA-coated tubes and screened for human immune cells using flow cytometry (LSR-II FACS analyzer, BD Biosciences, Mountain View, CA, USA). The CD45 percentage in humanized mice used in this study ranged from 25 to 50%. About 5% of our Hu mice develop graft vs.-host disease (GVHD) and these mice were excluded from our study.

### Infection of Hu-Mice With HIV-1

Twenty weeks after humanization, 12 Hu-mice were infected intraperitoneally (IP) with 2 × 10^4^ tissue culture infectious dose 50 (TCID_50_) of HIV-1_ADA_ (a macrophage tropic viral strain) (61–63). An additional six Hu-mice served as uninfected aged-matched controls. Peripheral blood samples were collected every four weeks via submandibular vein bleeding to assess HIV-1 viral RNA and to assess the dynamics of human immune cell markers by flow cytometric analysis. Plasma HIV-1 RNA levels were measured using an automated COBAS Ampliprep V2.0/Taqman-48 system (Roche Molecular Diagnostics, Basel, Switzerland) as per the manufacturer's instructions. The detection limit after dilution factor adjustment was 200 viral RNA copies/mL.

### Glo1 Gene Transfer to HIV-1 Infected Hu-Mice

Five weeks post-HIV-1 infection, Hu mice were divided into two groups. Animals in Group-1 received a single intravenous injection of AAV2/9-Endo-Glo1 (1.7 × 10^12^ virion particles/kg in sterile physiologic saline solution), while animals in Group-2 received saline (58, 60). Uninfected animals were injected with saline and were kept for the whole duration of the study (4 months total). The dose (multiplicity of infection) of AAV used in this study was selected from our earlier (60) and other previous studies (76, 77). AAV2/9 was selected because of its high tropism for cardiac myocytes and endothelial cells (76, 77). The endothelin-1 promoter was used to induce expression of Glo1 in these cells under inflammatory conditions.

### Assessment of Longitudinal Cardiac Function Using Echocardiography

Transthoracic conventional echocardiography was performed using a Fujifilm VisualSonics Vevo 3100 system (Fujifilm VisualSonics, Toronto, ON, CAN), employing a MX550D transducer with a center frequency of 40 Hz and an axial resolution of 40 μM, prior to, 5 and 16 weeks after infection with HIV-1 or saline injection (63). For this, hair on chests of mice were removed (Nair, Church & Dwight Co., Inc. NJ, USA). Twenty-four hours later, mice were anesthetized with 1–2% isoflurane (Cardinal Health, Dublin OH, USA) and taped in the supine position on a heated 37°C pad. Anesthesia was maintained with 0.5–3% isoflurane via a nose cone. Feet of mice were connected to ECG leads, and pulsed-wave Doppler images were acquired in the apical four chamber view with appropriate stage tilt and probe tilt to acquire maximum flow and digitally stored in cine loops. The offline Program Vevo LAB 3.1.1 was then used to assess peak early- and late-diastolic transmitral velocities (E and A waves), E-wave deceleration time, isovolumetric relaxation time (IVRT), isovolumetric contraction time (IVCT), mitral valve ejection time (MV ET), aortic ejection time (AET), and no flow time (NFT) as indices of diastolic function. E/A ratio was also calculated. Early diastolic tissue relaxation velocity (E') was measured using tissue Doppler, and E/e′ ratio was calculated. M-mode images were acquired from parasternal short-and long-axes views. Parameters measured include, left ventricular end-diastolic diameter (LVEDD), left ventricular end-systolic diameter (LVESD), left ventricular anterior wall thickness-diastole (LVAW;d), left ventricular anterior wall thickness-systolic (LVAW;s), left ventricular posterior wall thickness-diastolic (LVPW;d), posterior wall thickness-systolic (LVPW;s), mass, fractional shortening (FS), and ejection fraction (EF). Early diastolic tissue relaxation velocity (E') was measured using tissue Doppler, and E:e′ ratio was calculated. All ultrasound imaging and analyses were done in a blinded manner then decoded for statistical evaluation.

### Speckle Tracking

Parasternal long-axis and short axis B-mode echocardiographic images were obtained at a rate of >300 frames/second using the Fuji VisualSonics Vevo 3100 system and digitally stored in cine loops (60). Vevo LAB 3.1.1 was used to determine global longitudinal, radial, and circumferential strain using three to four consecutive cardiac cycles. The Vevo Strain Software was used to determine longitudinal, radial strain/strain rates, dyskinesis, and dyssynchrony during systole using six segment (anterior base, AB; anterior middle, AM; anterior apex, AP, posterior base, PB; posterior middle, PM; and posterior apex PA) analyses. All analyses were done in a blinded manner but decoded for statistical analyses.

### Microvessels Perfusion and Permeability in Cardiac Tissue

One week after the last echocardiographic measurement (17 weeks post-infection), half of mice from each group were injected with fluorescein isothiocyanate-labeled bovine serum albumin (FITC-BSA 40 mg/kg in sterile 1X PBS buffer, 50 μL) via tail vein (58, 60) and was allowed to circulate for 10 min, after which animals were anesthetized with 5% isoflurane. Chest cavities were opened and hearts were quickly removed and immersed in 4% paraformaldehyde for 24 h at 4°C. Hearts were then cut longitudinally into three sections, and the right 1/3 was transferred to 4% paraformaldehyde/15% sucrose solution for 24 h, followed by 4% paraformaldehyde/30% sucrose solution for 24 h, and then 30% sucrose solution for 24 h. Cryoprotected hearts were cut into 20 μm thick longitudinal/coronal sections on a microtome (Leica EM-UC 6, Leica Microsystems, Wien, Austria) and mounted onto pre-cleaned glass slides. Cardiac sections were then washed three times with 1X PBS to remove cutting medium. Vectashield™ mounting medium containing DAPI was added to the sections, and slides were cover slipped and dried overnight. Next day slides were placed on the head stage of a Nikon TE2000 microscope attached to a Coolsnap HQ2 CCD camera (Photometrics, Tuscon AZ, USA) and images were collected to assess the density of microvessels perfused with BSA-FITC and microvascular leakage. Analyses were done in a blinded manner but decoded for statistical evaluation.

### Masson-Trichrome and Immunofluorescence Staining in Cardiac Tissue

The left longitudinal 1/3rd of hearts from Hu-mice were also placed in 4% paraformaldehyde for 24 h and then processed and embedded in paraffin as described earlier (63). Five micrometer sections were then cut and placed onto glass slides. Slides were de-paraffinized with xylene (3 changes, ten minutes each) and rehydrated in decreasing concentrations of ethanol (100, 95, 70% and distilled water, three minutes each) followed by a saline wash. Masson Trichrome staining was conducted using rehydrated sections without modification to assess fibrosis (Sigma-Aldrich, St Louis, MO, USA). Sections were then cover slipped with Prolong Gold Anti-fade reagent. Images were then taken with a Nikon inverted fluorescence microscope (TE 2000) equipped with a CoolSNAP HQ2 CCD Camera (Photometrics, Tucson, AZ, USA). Image analysis software quantitated changes. This was completed in a blinded manner then decoded for statistical evaluation. Immunofluorescence staining was performed on cardiac tissues from Hu-mice (uninfected, HIV-1-infected and HIV-1-infected and treated with AAV2/9-Endo-Glo1) and from autopsied cardiac tissues obtained from uninfected and HIV-1 seropositive persons to determine the levels of the MG (hydroimidazolone isomer 1), Glo1, CD31 (measure of endothelial cell) and VAP-1 (a non-selective, inflammation-induced protein). Calponin-related protein (SM22α), a marker of contractile smooth muscle cells, served as reference to define microvessels. Primary antibodies were used at concentrations of 1:100 to 1:200 while secondary antibodies concentrations were 1:250 to 1:500. Horse serum (10%) was used as the blocking agents to reduce non-specific interactions. Images were taken with a Nikon inverted fluorescence microscope (TE 2000). Nikon Elements image analysis software was used to quantitate changes of VAP-1, MG-H1 and Glo1 immunoreactivities using 20× frames.

### MG and Semicarbazide-Sensitive Amine Oxidase (SSAO) in Plasma

MG levels (as surrogate MG-H1, OxiSelect™ Methylglyoxal Competitive ELISA, Cell Biolabs Inc, San Diego CA) in plasma were measured from Hu-mice (6 and 16 weeks, uninfected, HIV-infected and AAV2/9-Endo-Glo1-treated), and from uninfected healthy controls and PLWH as per manufacturers' instruction inside a BSL2+ Facility at UNMC. The activity of the non-selective inflammation marker, semicarbazide-sensitive amine oxidase (the soluble form of VAP-1) in plasma from Hu-mice was measured from (uninfected, HIV-infected and AAV2/9-Endo-Glo1-treated), and from uninfected healthy controls and PLWH using Fluoro-SSAO™ (Cell Technology, Mountain View CA) as per manufacture's instruction.

### Glo1 and VAP-1

Glo1 and VAP-1 levels in ventricular homogenates were also determined using Western blot assays (59). For this, cardiac tissues (50 mg) were chopped into small pieces, placed into 200 μL of cell lysis buffer (MicroRotofor Cell Lysis Kit (mammals), BioRad Inc., Burlingame CA) and sonicated 3 × 3 s with 10 s intervals on ice in between. Samples were then centrifuged at 3,000 × g for 5 min and the supernatants were collected and protein concentration was determined using Bradford Protein Assay Kit (BioRad Inc., Burlingame CA). Western blots were then carried out as described in earlier publications. Primary antibody concentrations were used at 1:1,000 dilutions and incubated for 16 h at 4°C and secondary antibody were used at 1:2,00 and incubated for 2 h at room temperature. β-actin served as the internal control to correct for variations in sample loading.

### Statistical Analyses

Data were analyzed using GraphPad Prism 7.0 software (La Jolla, CA) and presented in text as the mean ± the standard error of the mean. All experiments listed in this manuscript were performed using a minimum of three biologically distinct replicates. One-way ANOVA with Bonferroni correction for multiple comparisons were used. For studies with multiple time points, two-way factorial ANOVA and Bonferroni's *post-hoc* tests for multiple comparisons were performed. Studies were from six animals per group. Significant differences were determined at *p* < 0.05.

## Data Availability Statement

The original contributions presented in the study are included in the article/Supplementary Material, further inquiries can be directed to the corresponding author/s.

## Ethics Statement

The studies involving human participants were reviewed and approved by Human CD34+ Hu-NSG were isolated from umbilical cord blood obtained from UNMC labor and delivery department at UNMC with written consents from adult parents to use the remaining or discarded biological material for research. Samples were collected without identifiers under UNMC Institutional Review Board (IRB) exempt. The UNMC institutional IRB determined that these studies using anonymized cord blood samples doesn't constitute human subject research as defined at 45CFR46.102(f). We regularly collect the cord blood samples and isolate CD34+ hematopoietic cells which are either injected immediately into mice or stored in liquid nitrogen for future human reconstitution. The patients/participants provided their written informed consent to participate in this study. The animal study was reviewed and approved by University of Nebraska Medical Center (UNMC) Institutional Animal Care and Use Committee (IACUC). All animal procedures are approved under the IACUC protocols 18-110-08 and 10-107-01 for ECHO procedures. Written informed consent was obtained from the individual(s) for the publication of any potentially identifiable images or data included in this article.

## Author Contributions

KB conceived the experiments and planned along with all other authors. SG created humanized mice. PD, FA, BH, and KB conducted echocardiography and histopathological assays and interpreted the data sets for the humanized mice. PD and KB performed molecular, virologic and immunological studies, and related data analyses. JC, HF, and BM assisted with obtaining the de-identified human plasma and tissues. KB, EM, and SG performed and analyzed the microvascular permeability and fibrosis tests and analyses. FA, PD, JM, JC, BM, HG, HF, and SG interpreted the data and wrote the manuscript with editing. All authors contributed to the article and approved the submitted version.

## Funding

This work was supported in part by a pilot project from UNMC Center for Chronic HIV infection and Aging in NeuroAIDS (NIH P30 MH062261) and R56 HL151602-01A1.

## Conflict of Interest

The authors declare that the research was conducted in the absence of any commercial or financial relationships that could be construed as a potential conflict of interest.

## Publisher's Note

All claims expressed in this article are solely those of the authors and do not necessarily represent those of their affiliated organizations, or those of the publisher, the editors and the reviewers. Any product that may be evaluated in this article, or claim that may be made by its manufacturer, is not guaranteed or endorsed by the publisher.

## Supplementary Material

Supplementary Figure S1 Time frame of observation of immune status of animals used in the study.

Supplementary XLSX Showing (Sheet-1)—Patient data details from whom Plasma is analyzed. (Sheet-2)—Patient data details from whom autopsied tissues were used for the study.

Video 1 Uninfected control Hu-mice Video Short axis.

Video 2 Uninfected control Hu-mice Video Long axis.

Video 3 HIV-1 infected Hu-mice Video Short axis.

Video 4 HIV-1 infected Hu-mice Video Long axis.

Video 5 HIV-1 infected and AAV2/9-Endo-Glo1 injected mice Video short axis.

Video 6 HIV-1 infected and AAV2/9-Endo-Glo1 injected mice Video Long axis.
