# Supplementary material for: A Link Between Methylglyoxal and Heart Failure During HIV-1 Infection
Source: Front Cardiovasc Med. 2021 Dec 14;8:792180. doi: 10.3389/fcvm.2021.792180 (PMC8712558; doi:10.3389/fcvm.2021.792180)
Supplement: Supplementary Figure S1 — Time frame of observation of immune status of animals used in the study. [file Data_Sheet_1.PDF]

A Link Between Methylglyoxal and Heart Failure During HIV-1 Infection

Prasanta K Dash[1*], Fadhel A. Alomar[2*], Jesse L. Cox[3], JoEllyn McMillan[1], Bryan T. Hackfort[4], Brenda Morsey[5], Howard S. Fox[5], Howard E. Gendelman[1], Santhi Gorantla[1], and Keshore R. Bidasee[1,6,7]

Departments of [1]Pharmacology and Experimental Neuroscience, [3]Pathology and Microbiology, [4]Cellular and Integrative Physiology, [5]Neurological Sciences and [6]Environment and Occupational Health, University of Nebraska Medical Center, Omaha, NE 68198, [2]Department of Pharmacology, Clinical Pharmacology Imam Abdulrahman bin Faisal University, University of Dammam, Kingdom of Saudi Arabia and [7]Nebraska Redox Biology Center, Lincoln NE.

* Contributed equally

Correspondence

Keshore R. Bidasee, Ph.D.

Department of Pharmacology and Experimental Neuroscience, 985800 Nebraska Medical Center, Durham Research Center, DRC 3047, Omaha, NE, USA. Tel: (402) 559-9018; Fax: (402) 559-7495, E-mail: kbidasee@unmc.edu.

Supplemental Figure with Legend

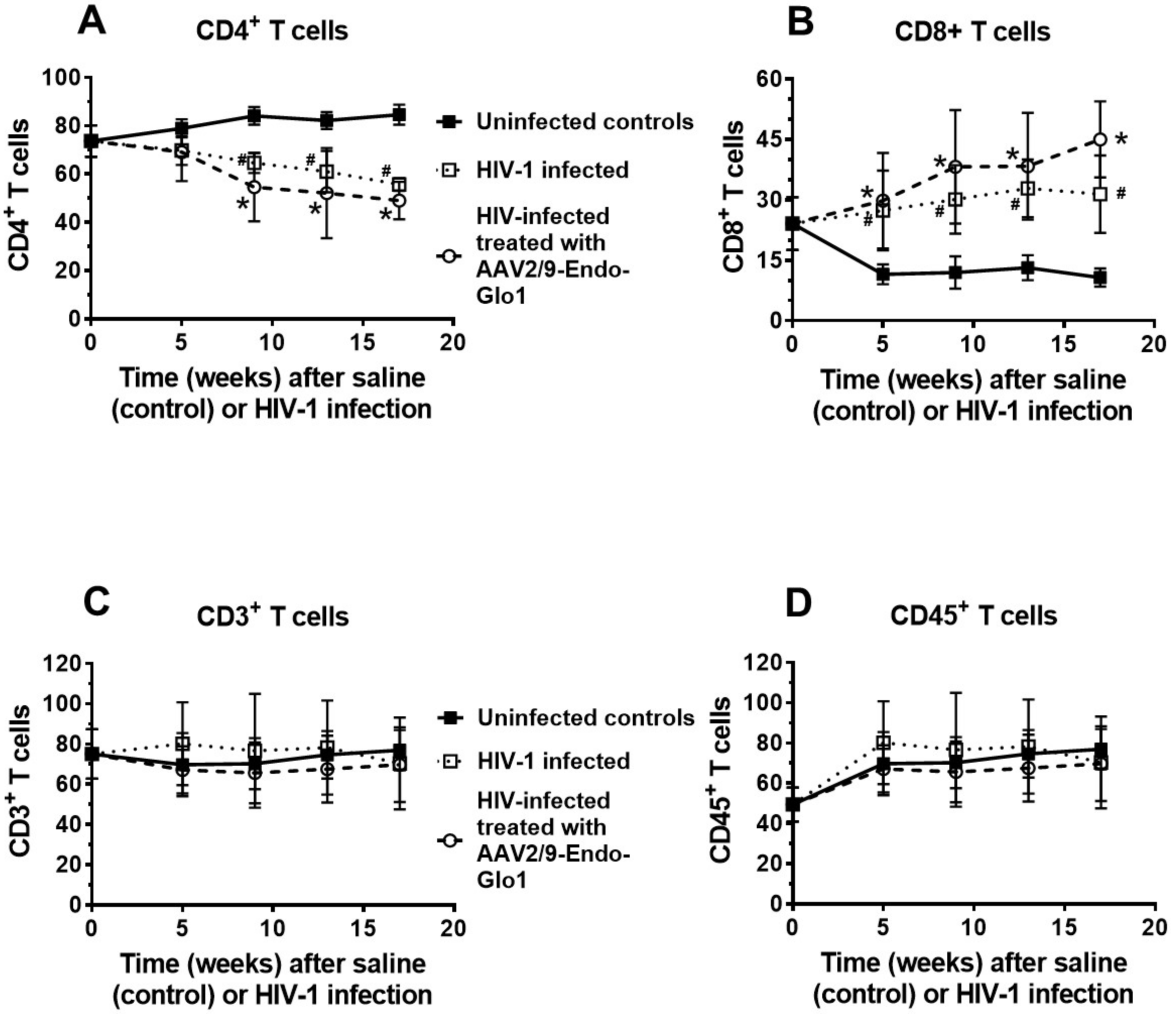

Supplemental Fig. S1: Time frame of observation of immune status of animals used in the study. Graphs A and B show longitudinal CD4+ and CD8+ cell populations in peripheral blood from uninfected Hu-mice and HIV-1-infected Hu-mice. Graphs C and D show longitudinal CD3+ and CD45+ cell populations in peripheral blood from uninfected Hu-mice and HIV-1-infected Hu-mice. Data shown on graphs are mean ± SEM from n≥6 mice per group. *denotes significantly different from uninfected Hu-NSG mice ($p<0.05$). $^{\#}$denotes significantly different from HIV-1 infected Hu-mice ($p<0.05$).
